# Better care for babies: the added value of a modified reverse syphilis testing algorithm for the treatment of congenital syphilis in a maternity Hospital in Central African Republic

**DOI:** 10.1186/s12887-019-1622-4

**Published:** 2019-08-15

**Authors:** Oluwakemi F. Ogundipe, Rafael Van den Bergh, Behounde Thierry, Kudakwashe C. Takarinda, Claude P. Muller, Collins Timire, Severine Caluwaerts, Pascale Chaillet, Isabel Zuniga

**Affiliations:** 1 Médecins Sans Frontières, Operational Centre Brussels, Brussels, Belgium; 2 Ministry of Health, Bangui, Central African Republic; 3 International Union Against Tuberculosis and Lung Disease, Paris, France; 4 Ministry of Health and Child Care, AIDS and TB Department, Harare, Zimbabwe; 5 Luxembourg Institute of Health, Esch-Alzette, and Laboratoire National de Santé, Dudelange, Grand Duchy of Luxembourg; 6 International Union Against Tuberculosis and Lung Disease, Harare, Zimbabwe

**Keywords:** Congenital, Syphilis, Non-treponemal, Operational research, Central African Republic, Low resource

## Abstract

**Background:**

In high syphilis prevalence settings, the syphilis testing and treatment strategy for mothers and newborns must be tailored to balance the risk of over treatment against the risk of missing infants at high-risk for congenital syphilis. Adding a non-treponemal test (Rapid Plasma Reagin - RPR) to a routine rapid treponemal test (SD Bioline Syphilis 3.0) for women giving birth can help distinguish between neonates at high and low-risk for congenital syphilis to tailor their treatment. Treatment for neonates born to RPR-reactive mothers (high-risk) is 10 days of intravenous penicillin, while one dose of intramuscular penicillin is sufficient for those born to RPR non-reactive mothers (low-risk). This strategy was adopted in March 2017 in a Médecins Sans Frontières supported hospital in Bangui, Central African Republic. This study examined the operational consequences of this algorithm on the treatment of newborns.

**Methods:**

The study was a retrospective cohort study. Routine programmatic data were analysed. Descriptive statistical analysis was done. Total antibiotic days, hospitalization days and estimated costs were compared to scenarios without RPR testing and another where syphilis treatment was the sole reason for hospitalization.

**Results:**

Of 202 babies born to SD Bioline positive mothers 89 (44%) and 111(55%) were RPR-reactive and non-reactive respectively (2 were unrecorded) of whom 80% and 88% of the neonates received appropriate antibiotic treatment respectively. Neonates born to RPR non-reactive mothers were 80% less likely to have sepsis [Relative risk (RR) = 0.20; 95% Confidence interval (CI) = 0.04–0.92] and 9% more likely to be discharged [RR = 1.09; 95% CI = 1.00–1.18] compared to those of RPR-reactive mothers. There was a 52%, and 49% reduction in antibiotic and hospitalization days respectively compared to a scenario with SD-Bioline testing only. Total hospitalization costs were also 52% lower compared to a scenario without RPR testing.

**Conclusions:**

This testing strategy can help identify infants at high and low risk for congenital syphilis and treat them accordingly at substantial cost savings. It is especially appropriate for settings with high syphilis endemicity, limited resources and overcrowded maternities. The babies additionally benefit from lower risks of exposure to unnecessary antibiotics and nosocomial infections.

## Background

Syphilis is a bacterial infection caused by *Treponema pallidum*. The prevalence of syphilis in pregnant women is < 1% worldwide and 1.5% in the African region; Africa carries 57% of the global burden of syphilis infection in pregnancy [1]. During pregnancy, mother-to-child transmission (MTCT) rates for active syphilis could range from 60 to 100% [2]. Adverse outcomes occur in 52% of pregnancies with probable active syphilis, and include perinatal deaths neonatal deaths and prematurity or low birth weight [1, 3, 4]. The neonatal mortality rate from congenital syphilis can be as high as 10% and long-term morbidity can also be substantial [3]. The African region also accounts for 61% of the global burden of syphilis-associated adverse outcomes in children [1]. These adverse outcomes are preventable if adapted diagnostic and treatment algorithms are implemented.

The two types of tests that are typically available for syphilis testing are treponemal and non-treponemal tests. The treponemal tests, while specific, do not distinguish between past and active infection. The non-treponemal tests can have false positives due to other spirochetal and viral infections, malaria, TB and other conditions [2, 5]. Individually, neither type of test can definitively diagnose active syphilis, so a combination of both tests [3, 6] is typically recommended to properly establish a diagnosis. Probable active syphilis is thus defined as both a non-treponemal and treponemal test being positive. The classic testing strategy is a screening test with a non-treponemal test followed by a confirmatory test with a treponemal test. Depending on these results further testing with a non-treponemal test may be required based on the results of the first two tests. The reverse testing strategy starts with a treponemal test followed by a confirmatory non-treponemal test with further treponemal testing based on the results of the first two tests.

The diagnosis of congenital syphilis is particularly difficult because testing algorithms for delivering women using these two types of tests cannot definitively confirm MTCT [5]. Babies born to mothers with syphilis (diagnosed by either test type) are classified into different risk categories, and additional testing of the neonate is recommended in some guidelines [2, 6]. Neonatal treatment is a 10-day course of intravenous penicillin when the risk for congenital syphilis is high or a single intramuscular injection of penicillin when the risk is low. Neonatal risk depends on the testing algorithm, maternal history and previous maternal treatment for syphilis [2, 3, 6].

In high-prevalence and low-resource settings (which often coincide) where there is a higher chance of maternal past infection, which carries a low-risk of congenital syphilis [5], the syphilis testing strategy needs to be tailored. In settings where syphilis testing is not guaranteed during antenatal care (ANC), syphilis testing at the time of delivery is warranted. It must balance the risk of over treatment, which can lead to congested neonatal wards, and unnecessary exposure to antibiotics, with the risk of missing infants that are at a higher risk for congenital syphilis. One such setting is the Central African Republic (CAR), which has the second highest neonatal mortality in the world at 41.5 per 1000 live births [7, 8]. The prevalence of syphilis in pregnancy is estimated between 4.7 to 10% [8, 9]. Along with 11 other countries, CAR has been targeted by the World Health Organization (WHO) for intensified support for the elimination of MTCT of syphilis [9]. Other syphilis testing strategies such as paired sera testing during pregnancy are not part of the testing algorithm for syphilis in CAR. Instead, single Venereal Disease research laboratory (VDRL) or Treponema pallidum hemagglutination assay (TPHA) are recommended, because ANC attendance, after the initial visit, is unreliable [10, 11]. Polymerase chain reaction (PCR) testing is also not available in CAR, even at the highest level of care at the district hospitals [10].

In the Medecins Sans Frontieres (MSF) supported Castors Maternity Hospital in CAR, maternal syphilis was diagnosed using the SD Bioline Syphilis 3.0- Standard Diagnostics (treponemal) test until March of 2017. Since reliable maternal treatment information was not available, it was not possible to differentiate high and low risk neonates. As a result all infants born to SD Bioline positive mothers were treated for 10-days with IV penicillin. This potentially resulted in over-treatment as neonates born to mothers with past infection were not excluded. In March of 2017, the Rapid Plasma Reagin (RPR) (non-treponemal) test was added as a confirmatory test to distinguish between past and probable active infection. The mothers and babies were treated based on the results of the two tests in this modified reverse testing algorithm. This allowed the option of giving infants at low-risk for congenital syphilis a single dose of penicillin instead of a 10-day treatment course [2, 6]. This change was expected to reduce unnecessary antibiotic exposure and to reduce the number of infants staying for 10 days in the typically crowded neonatal ward.

Several studies have investigated the cost-effectiveness of syphilis testing in avoiding adverse outcomes for both the mothers and infants, while others have looked at the effect of different maternal testing strategies on the clinical management of mothers and the elimination of MTCT in various settings [12–16]. These analyses are based on theoretical models and projections [12–14]. Yet, no study has fully explored the operational benefits of using this type of testing/treatment algorithm (in this case: treponemal test + non-treponemal test) for the initial hospital care of newborns. We therefore assessed the effect of such an algorithm on hospitalization days and associated costs for neonatal patients in this high-prevalence setting.

## Methods

### Aim

We set out to determine among all delivering mothers at Castors Comprehensive Emergency Obstetric and Newborn Care Centre (CEmONC) from 23 March 2017–11 February 2018: i) the number and proportion of mothers testing positive for syphilis infection using SD Bioline test and their RPR results ii) the proportion of neonates receiving appropriate antibiotic therapy based on the maternal RPR results iii) their hospitalization outcomes iv) actual antibiotic and hospitalization days by treatment regimen and v) treatment regimen-related health service costs.

### Study design

Retrospective analytical cohort study using routinely collected programme data.

### General setting

CAR is located in central Africa. It has an estimated population of 4.9 million [17]. Since 2013, there have been recurrent internal conflicts leading to large population displacements in and around Bangui, the capital city. In May 2015 there was a peace agreement that was signed by 9 of the 10 then existing militia groups. Nevertheless, armed conflicts continue intermittently. Currently 14 of the 17 prefectures of the country are under the control of armed groups that limit free movement throughout most of the country [18].

### Specific setting

Bangui has an estimated population of 851,000 [18, 19] and is divided into 8 zones. The study setting, Castors Maternity Hospital is located in the most densely populated 5th zone. This hospital has been supported since June 2014 by MSF Operational Centre Brussels, an international non-governmental organization providing humanitarian aid and free medical assistance.

At Castors, MSF initially opened a Basic Emergency Obstetric and Newborn Care centre (BEmONC) but due to the patient needs it evolved into a CEmONC with management of obstetrical complications and neonatal services, comprised of kangaroo mother care and a neonatal intensive care unit, providing care for babies born in this facility. MSF does not provide or support ante-natal care consultations in this setting. In the last 3 years, bed occupancy rates have exceeded the capacity of the structure with both women and newborns being cared for in crowded facilities that challenge adequate infection prevention and control measures. Neonatal admissions for a 10-day course of IV penicillin for risk of congenital syphilis contribute to this congestion.

### Syphilis testing and treatment

Pregnant women delivering in Castors Maternity Hospital were tested for syphilis at the time of or after delivery. Reliable information about prior syphilis infection could not usually be obtained. Any history of syphilis infection and/or treatment was solely based on self-reporting at the time of the delivery as women did not typically have an accessible patient file with this information. In March 2017 a new algorithm was introduced for testing mothers and treating newborns delivered at Castors with the introduction of Rapid Plasma Reagin (RPR) as a non-treponemal screening test.

Under this new algorithm, high risk of congenital syphilis was defined as a maternal RPR titre equal or greater than 1:4, or evidence of maternal re-infection (e.g., a chancre on physical examination), or a neonatal physical examination consistent with congenital syphilis (see Table 1). Low risk of congenital syphilis was defined as a maternal titre of 1:2 without any evidence of maternal re-infection and a newborn exam that was not indicative of congenital syphilis. Neonatal RPR testing, neonatal cerebrospinal fluid examination and additional tests such as complete blood counts and bone radiographs were not routinely done.

**Table 1:** Clinical features of early congenital syphilis

Hepatosplenomegaly.	
Jaundice.	
Nasal discharge (snuffles).	
Lymphadenopathy.	
Maculopapular rash.	
Skeletal abnormalities (Osteochondritis, Periostitis).	
Pneumonia.	
Pseudoparalysis.	
Oedema.	
Mucocutaneous lesions.	
Hemolytic anaemia.	
Thrombocytopenia.	

All clinical features above are present at birth or within 4–8 weeks after birth

**Fig. 1:**
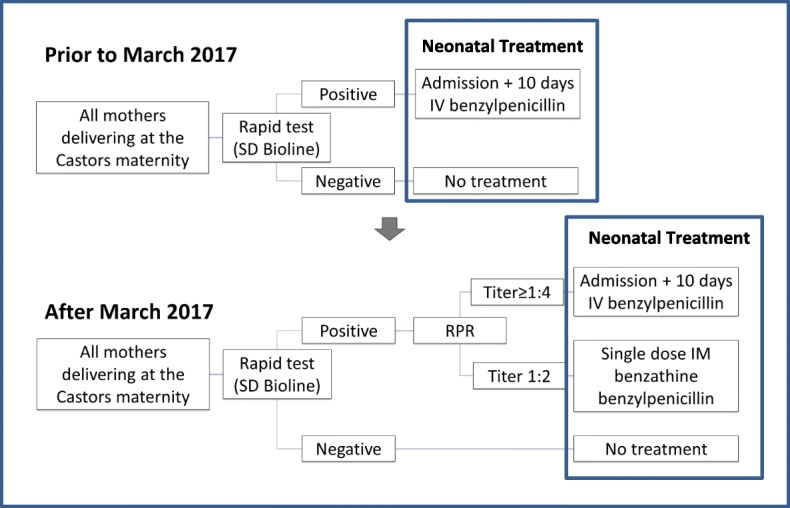
Algorithm for treating neonates with risk of congenital syphilis before and after March 2017. RPR = Rapid plasma reagin test. SD Bioline and RPR tests are done on the mothers and the infants are treated based on the results for risk of congenital syphilis at MSF Castors Maternity Hospital

The algorithms used for testing and treating neonates based on maternal syphilis tests are shown in Fig. 1. The new testing algorithm was a modified version of the reverse testing algorithm where no further treponemal testing of the mother or the neonate was done after the confirmatory non-treponemal test. The delivering mothers who were positive for syphilis by the SD Bioline Syphilis 3.0 test were treated with 3 doses of intramuscular penicillin weekly regardless of RPR result, unless there was evidence that the mother was treated before and there was no suspicion of recent re-infection.

The SD Bioline Syphilis 3.0 (Standard Diagnostics) is a solid phase immunochromatographic assay for the qualitative detection of antibodies of all isotypes (IgG, IgM, IgA) against *Treponema pallidum* (TP) with a manufacturer-reported sensitivity and specificity of 99.3 and 99.5% respectively. The RPR (ELITech group, France) is a serologic cardiolipid and non-treponemal test for the rapid diagnosis of syphilis with > 99% sensitivity and specificity according to the manufacturer. Both tests are done in the Castors laboratory, a relatively well-stocked laboratory with the capacity to perform serological tests.

### Study population

All mothers who were positive for syphilis by SD Bioline test and their live newborns that were tested and treated for syphilis from March 23rd 2017 to February 11th 2018.

### Data variables, sources of data and data collection methods

Variables collected included aggregate number of live births during the study period, date and result of RPR tests for mothers who were SD Bioline positive, maternal age, and neonatal information on date of birth, birthweight, gender, admission date, number of days of penicillin received, exit diagnoses, exit date and disposition. Data on unit costs of the RPR test and penicillin as well as hospitalization costs were also obtained. Antibiotic days were defined as the aggregate number of days for which any amount of a specific antibiotic was administered to a patient and documented in the medical records. Hospital days were defined as the total number of days a patient stayed in hospital. Admissions for < 24 h were counted as one hospital day.

Maternal and neonatal data were abstracted from project monthly situation reports and annual and trimestral reports, laboratory records, neonatal register and neonatal patient files. Only data on the mother’s age were available from these sources. No other sociodemographic data (e.g. Profession, parity, number of living children, and psychosocial risk factors) were available. Costs of daily maternal room and board costs were obtained from the project supply manager whilst program data on antibiotic and medical test costs were obtained from the project pharmacist. Data were collected during a routine support visit in June 2018.

Patient chart numbers were collected in place of names during data abstraction and merged to sequential numbers to create unique identifiers. The data were collected on paper collection forms and afterwards double entry was done into an EpiData database (Version 3.1 EpiData Association, Odense, Denmark).

### Analysis and statistics

Data analysis was done using EpiData Analysis V2.2.2.186 (EpiData Association, Odense, Denmark) and Stata MP 15.1 (StataCorp, College Station, Texas 77845 USA). Means (standard deviations) were calculated for normally distributed continuous data and medians (interquartile ranges) for skewed continuous data whilst numbers and proportions were calculated for categorical data. Differences between groups were assessed using Pearson’s *X*^2^ (Chi-square) test, and alternatively the Fisher’s exact test if assumptions for the Chi-square test were not met. The level of significance was set at *p* ≤ 0.05 and 95% confidence intervals (CI) were used throughout.

For the health cost analysis, actual calculated costs were compared to counterfactual theoretical models described in Table 2 below. The formulas used for these calculations are also shown in Table 2. The costs of other treatments unrelated to the diagnosis of risk of congenital syphilis were not included in the cost calculations. The 8 cases where the length of stay or RPR titre or number of penicillin days was unknown were excluded from this calculation. A conversion factor of 655.08 CFA to 1 euro was used for calculations. The estimated cost per day of hospital stay took into account food and a hygiene kit (soap, cloth, mosquito net) provided to each mother (10.27 Euros). Staff time was not included in the cost calculation. The costs of an individual RPR test unit and the cost of a unit of penicillin were constant values obtained from the MSF catalogue.

## Results

**Fig. 2:**
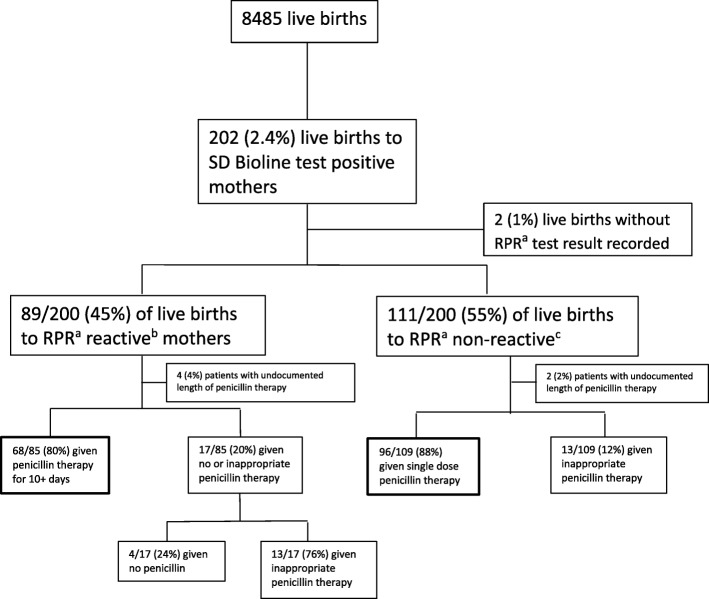
Pattern of deliveries and syphilis testing of mothers and treatment of their infants. Legend: SD Bioline and RPR tests are done on the mothers and the infants are treated based on the results at MSF Castors Maternity Hospital from March 2017 to February 2018.^a^ RPR = Rapid plasma reagin test^b^ RPR reactive = titre > 1:4^c^ RPR non-reactive = titre of 1:2. MSF = Médecins Sans Frontières

The number of total live births and the live births to mothers who were SD Bioline positive and subsequently RPR tested is shown in Fig. 2. Of the 8,485 live births at MSF Castors Maternity hospital during the study period, 202 (2.4%) were born to SD Bioline positive mothers of whom 200 (99%) had recorded RPR test results. The breakdown of newborns enrolled in the study shows that 80% of babies of RPR reactive mothers and 88% of neonates of RPR non-reactive mothers received appropriate treatment (*p* = 0.13). The neonates who received inappropriate treatments were those who did not receive the correct penicillin treatment based on maternal RPR results. Among the 17 babies born to RPR reactive mothers who received inappropriate treatment, 7 (41%) were discharged earlier than ten days while the other 10 had treatments interrupted by death, transfer or leaving against medical advice. No treatment was discontinued due to an allergic reaction. On the other hand all of the 13 babies who received inappropriate treatment among those born to RPR non-reactive mothers were discharged. All of these 13 babies received at least one day of treatment with penicillin.

**Table 2:** Estimation of Syphilis Treatment Associated Cost (Euros) for Babies Treated for Risk of Congenital Syphilis

Cost Calculations (Euros)^a^	
**Scenario A:** Assumes that all neonatal patients were treated for 10 days with penicillin (PCN) irrespective of maternal RPR results.	
*Sy*philis treatment associated cost of hospital stay for each patient = (Cost of unit of PCN [3.07] × 23 doses) + (Cost of hospital day [10.27] × 10 days).	
**Scenario B:** Assumes that all mothers were tested with both SD Bioline and RPR. Patients born to RPR reactive mothers were treated for 10 days with IV penicillin while patients born to RPR non-reactive mothers were treated with a single dose of penicillin.	
*RPR reactive:* Syphilis treatment associated cost of hospital stay = (Cost of unit of PCN [3.07] × 23 doses) + (Cost of hospital day [10.27] × 10 days) + Cost of RPR test unit [0.72].	
*RPR non-reactive:* Syphilis treatment associated cost of hospital stay = Cost of unit of PCN [3.07] + Cost of hospital day [10.27] + Cost of RPR test unit [0.72].	
**Actual situation:**	
Syphilis treatment associated cost of hospital stay for each patient = (Cost of unit of PCN [3.07] × # of doses of PCN) + (Cost of hospital day [10.27] × # of hospital days) + (Cost of RPR test unit [0.72]).	

*PCN* Penicillin, *RPR* Rapid plasma reagin

Legend: ^a^All costs are in Euros. Estimated and actual costs at MSF Castors Maternity Hospital, March 2017 to February 2018

Selected demographic characteristics of the neonates and maternal age are shown in Table 3. The average length of stay for babies born to RPR reactive mothers was 8.9 +/− 2.9 days, while the average length of stay for babies born to RPR non-reactive mothers was 2.13 +/− 4.9 days (*p* < 0.001).

**Table 3:** Demographic characteristics of neonates and their mothers stratified by Maternal RPR result

Demographic Characteristics	RPR^a^ Reactive^b^		RPR^a^ Non-reactive^c^		*p*-value
	*N* = 89	(%)	*N*=111	(%)	
Total	89	(100)	111	(100)	
Birth weight (g)					NS
<1500	0	(0)	2	(2)	
1500-2499	10	(11)	10	(9)	
> 2500	79	(89)	99	(89)	
Sex					NS
Male	41	(46)	55	(50)	
Female	46	(52)	56	(50)	
Not recorded	2	(2)	0	(0)	
Maternal age					
15-24	28	(31)	48	(43)	NS
25-34	39	(44)	37	(33)	NS
35-44	16	(18)	15	(14)	NS
> 45	0	(0)	1	(1)	NS*
Not recorded	6	(7)	10	(9)	

All neonates with risk of congenital syphilis MSF Castors Maternity Hospital from March 2017 to February 2018

*X*^2^ test used for all comparisons except where indicated as (*) Fisher’s exact test

^a^*RPR* Rapid plasma reagin test^b^ RPR reactive = titre > 1:4^c^ RPR non-reactive = titre of 1:2

*NS* Non-significant

Table 4 shows the additional diagnoses and outcomes of neonates born to RPR reactive and non-reactive mothers in the study period. Comparable proportions of babies born to RPR reactive and RPR non-reactive mothers had other discharge diagnoses (27% versus 26%, *p* = 0.87). The babies born to mothers who were RPR non-reactive were significantly less likely to have sepsis [RR = 0.20; 95% CI = 0.04–0.92] and were more likely to be discharged [RR = 1.09; 95% CI = 1.00–1.18, *p* = 0.049] than babies born to RPR reactive mothers. The mortality rate among all patients was 1.5%. Of the patients whose mothers were RPR reactive, there were 10 patients who were not discharged but instead were transferred, died or left against medical advice. These patients contributed a total of 29 days instead of the predicted total of 100 days in hospital.

**Table 4:** Diagnoses and hospitalization outcomes of newborns treated for risk of congenital syphilis

	RPR^a^ Reactive^b^		RPR^a^ Non-reactive^c^		*p-value*
	*N*=89	(%)	*N*=111	(%)	
Discharge Diagnoses^d^					
None	65	(73)	82	(74)	NS
Sepsis	8	(7)	2	(2)	0.02*
Perinatal asphyxia	4	(4)	7	(8)	NS*
Premature/low birth weight	3	(3)	3	(3)	NS*
Antibiotic prophylaxis (well baby)^e^	9	(8)	15	(17)	NS
HIV Exposure	4	(4)	9	(10)	NS
Number of diagnoses					
None	65	(73)	82	(74)	NS
One diagnosis	20	(22)	22	(20)	NS
Two diagnoses	4	(5)	7	(6)	NS*
Disposition					
Discharged	79	(89)	107	(96)	0.05
Transferred	2	(2)	0	(0)	NS*
Dead	2	(2)	1	(1)	NS*
Left against medical advice	6	(7)	3	(3)	NS*

*X*^2^ test used for all comparisons except where indicated as (*) Fisher’s exact test

Neonatal treatment was based on Maternal RPR Results at MSF Castors Maternity Hospital from March 2017 to February 2018

^a^*RPR* Rapid plasma reagin test ^b^ RPR reactive = titre > 1:4^c^ RPR non-reactive = titre of 1:2

^d^Sum of n for discharge diagnoses is not equal to 100% as patients can present with more than one diagnosis

NS = Not significant

^e^Antibiotic prophylaxis = infants who are treated for a minimum of 48 h of antibiotics based on maternal and birth risk factors but who are otherwise well

For the subsequent analyses two counterfactual scenarios were used. In scenario A the theoretical antibiotic treatment was based solely on the SD Bioline test (irrespective of the RPR result), while in scenario B patients born to RPR reactive mothers would receive 10 days of treatment and those born to RPR non-reactive mothers would receive 1 day of treatment, and no other factors affect treatment or length of stay. In scenario B, treatment for risk of congenital syphilis was considered to be the only condition for which the child was admitted.

**Fig. 3:**
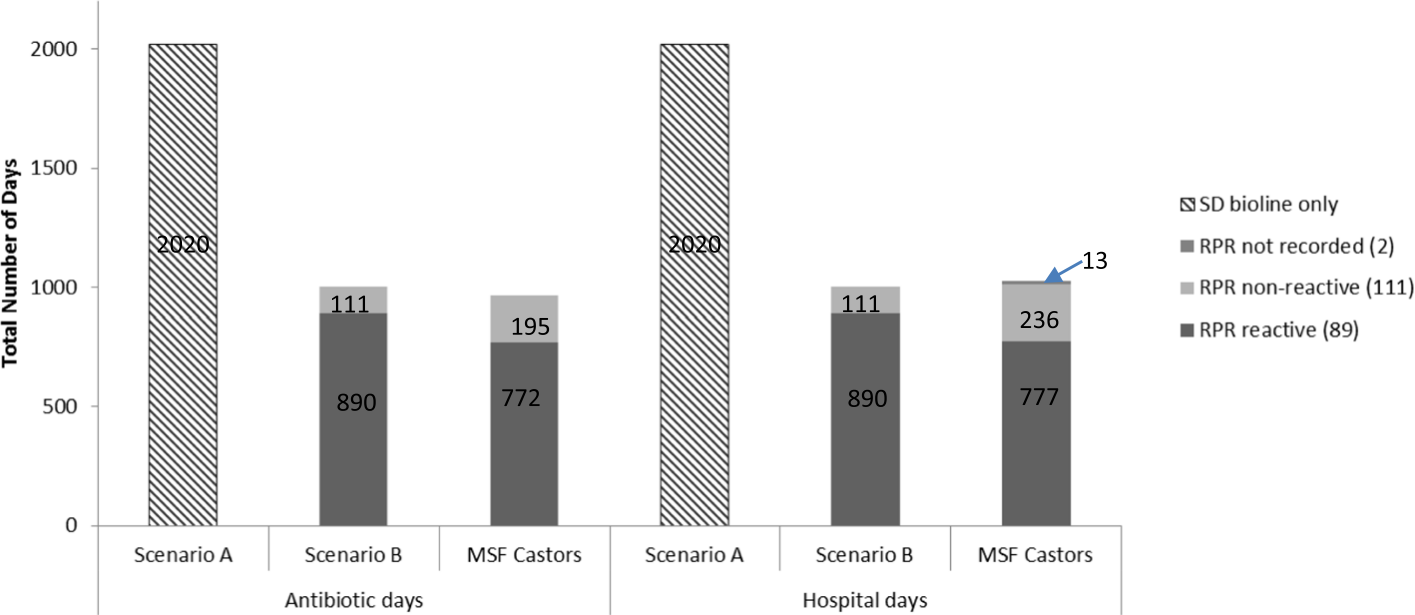
Actual and theoretical antibiotic and hospital days saved for neonates at MSF Castors Maternity Hospital. Legend: RPR = Rapid plasma reagin. Antibiotic days = Aggregate number of days for which any amount of a specific antibiotic is administered to a patient and documented in the medical record (adapted from CDC definition). Hospital days = The total number of days a patient stays in hospital. **Scenario A:** Assumes that all patients were treated for 10 days with penicillin irrespective of maternal RPR results. **Scenario B:** Assumes that all mothers were tested with both SD Bioline and RPR. Patients born to RPR reactive mothers were treated for 10 days with IV penicillin while patients born to RPR non-reactive mothers were treated with a single dose of penicillin. **MSF Castors:** Actual antibiotic and hospital days for patients born to RPR reactive and RPR non-reactive mothers at MSF Castors from March 2017 to February 2018

The actual total number of antibiotic and hospitalization days for neonates included in the study period as compared to the two counterfactual scenarios is shown below (Fig. 3). At Castors, the total number of days when the neonates were receiving penicillin for syphilis therapy accounted for 94% of the total hospitalization days. There was a 52% reduction in the total number of antibiotic days and a 49% reduction in the total number of hospitalization days when compared to scenario A. The actual total number of antibiotic days was 3% less than the theoretical antibiotic days predicted by scenario B.

**Fig. 4:**
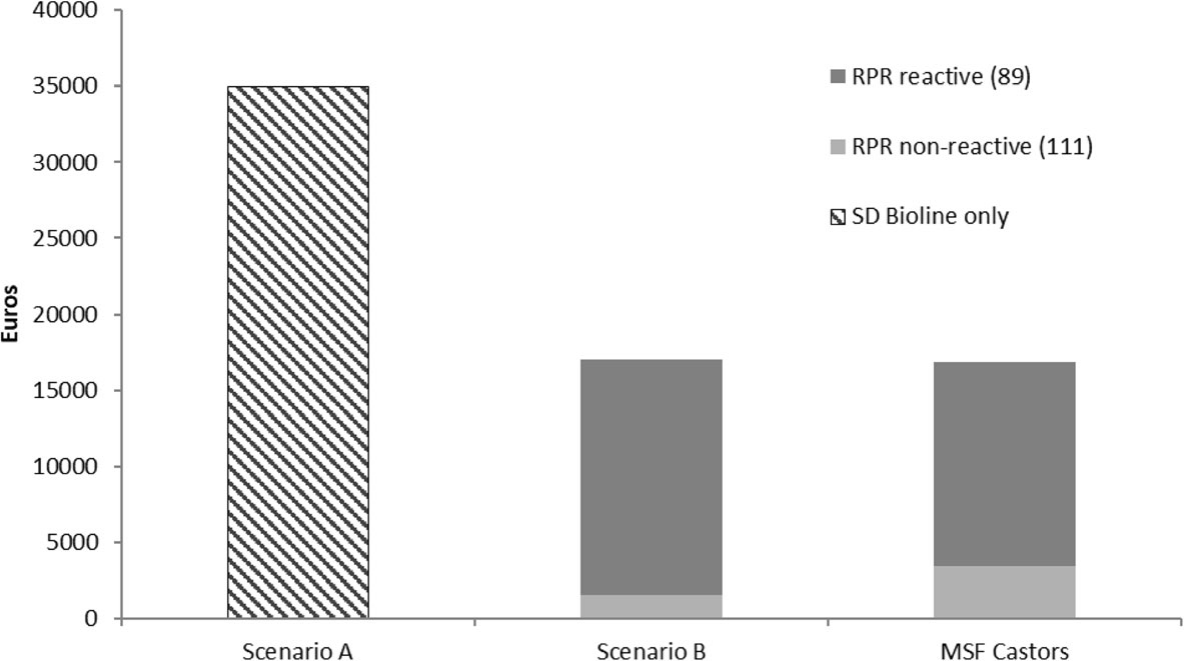
Health Cost Comparisons Between Actual and Theoretical Situations for Neonates at Risk for Congenital Syphilis. Legend: RPR = Rapid plasma reagin. For descriptions of ***Scenarios A*** and ***B*** see Legend of Fig. 4. ***MSF Castors***: Actual antibiotic and hospital days for patients born to RPR reactive and RPR non-reactive mothers at MSF Castors from March 2017 to February 2018

**Fig. 5:**
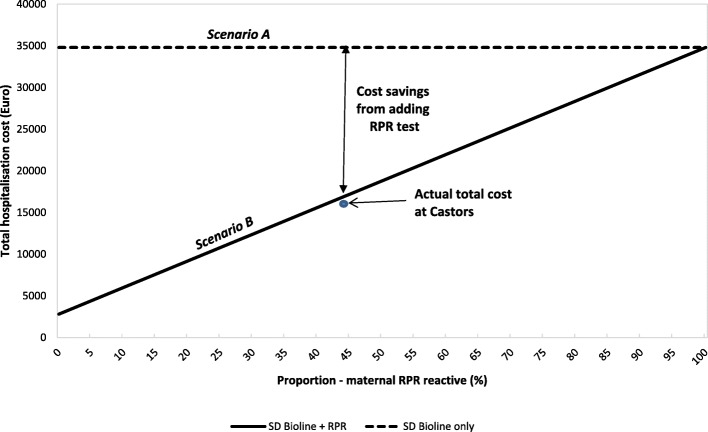
Health Cost Comparisons for neonates as a function of proportion of RPR reactive mothers. Legend: RPR = Rapid plasma reagin. All costs are estimated at MSF Castors Maternity Hospital from March 2017 to February 2018. For descriptions of ***Scenarios A*** and ***B*** see Legend of Fig. 4

Figures 4 and 5 compare the total cost for all patients included in the study and the theoretical costs in scenarios A and B described above. Hospitalization days and number of doses of antibiotics were used in this calculation. The analysis showed that the total cost of hospitalization during the study period was 52% lower than it would have been without RPR testing (scenario A). Meanwhile there was no significant difference between the total theoretical cost (scenario B) and the total actual cost (Actual cost was 1.3% less).

In scenario B, where cost was only dependent on maternal RPR results, neonates born to RPR non-reactive mothers should only account for 9% of the total cost, while at Castors these neonates account for 21% of the total cost (Fig. 4) due to additional hospitalization days for other causes.

## Discussion

In this study the effect on newborns of the introduction of a more comprehensive testing/ treatment algorithm for maternal syphilis was examined. This algorithm was a step up in laboratory testing and added some complexity to care for infants at risk for syphilis. Nevertheless, our results showed that its implementation had added value since all but 2 SD Bioline positive mothers failed to have recorded confirmatory RPR tests, and most of the infants were treated accordingly. Among the babies born to RPR reactive mothers, the percentage of babies treated adequately could have potentially been even higher than 80% if outcomes of transfer and leaving against medical advice were excluded. This demonstrates that this algorithm is suitable for high-prevalence settings that have the minimal laboratory capacity to prepare serum and have minimal human and monetary resources.

The frequency of cases with probable active infection was 1%, which is considerably less than the 4.7% most recently reported for CAR [8], and less also than the 4.4% estimate for the general population in Bangui from 2013 [20]. These differences may be due to the population shifts that occurred in Bangui since the conflicts (which also began in 2013). It may also be due to the location of the maternity in Bangui, an urban centre, with potentially increased access to health services when compared to other parts of the country. A study comparing syphilis rates among pregnant women in urban and rural CAR showed four-fold higher odds of having probable active syphilis in rural areas in CAR [21].

It is estimated that 16% of babies born to mothers with untreated syphilis would have congenital syphilis [22], with half of these babies showing clinical signs of low birth weight/prematurity and skeletal malformations [22]. We found neither clinical signs of neonatal syphilis (no diagnoses of congenital malformations), nor any association with prematurity/low birth weight in this study other than the increased likelihood of the babies born to RPR reactive mothers of being diagnosed with sepsis.

As expected the dual testing algorithm considerably reduced the number of infants requiring a 10-day treatment and halved the antibiotic and hospitalization days. The results also demonstrated that despite other clinical diagnoses and reasons for continuing hospital care, the antibiotic days and hospitalization days saved were close to what was predicted when risk of syphilis was the only diagnosis for which these babies were admitted. The actual total number of antibiotic days was less by 3% than the theoretical total antibiotic days predicted mostly due to patients who were transferred, left against medical advice or died before receiving the full course of treatment.

Notably 6 of the 17 babies born to RPR reactive mothers who did not receive adequate penicillin therapy left against medical advice. It is possible that a higher number of babies would have left early without receiving adequate treatment if this algorithm was not in place and all babies had to be treated with 10 days of penicillin. Another notable finding was that 8% (7/89) of babies born to RPR reactive mothers were treated inappropriately. This finding will be relayed back to Castors to reinforce their screening algorithm to assure that no further babies at high risk for congenital syphilis are discharged with inappropriate treatment. The 12% of babies born to RPR non-reactive mothers who were treated inappropriately is less concerning as all of them received at least one day of penicillin therapy, and a single (intramuscular) dose of penicillin is as effective as a classic 10 days of therapy for babies whose risk of syphilis is low [22]. The babies at low risk for syphilis benefited from treatment according to this algorithm as they had reduced exposure to unnecessary antibiotics.

The reduced antibiotic and hospital days also resulted in considerable cost savings of 52% because the RPR test is inexpensive. In other settings with a high prevalence of syphilis e.g. South Sudan, Mali [8], where funding and human resources are scarce, these cost savings could lead to improved quality of care and less crowded spaces for mothers and babies. This would allow more mothers and babies to be admitted and treated.

The reduction in hospitalization days had an added benefit of avoiding other risks of hospitalization such as nosocomial infections, medication errors etc. Castors Neonatal Ward had a nosocomial outbreak of *extended spectrum beta lactamase* (ESBL) *Klebsiella species* during the observation period. Babies who spent only one day hospitalized for treatment for risk of syphilis instead of ten reduced their risk of nosocomial infection. With the rise of ESBL and other resistant forms of neonatal sepsis in low and middle-income countries [23] an algorithm like this will contribute to reducing the risk of nosocomial infections with resistant pathogens in the hospitalized neonatal population. This effect would be much greater in the low income countries that have low ANC coverage and limited antenatal screening and treatment for syphilis [11].

This study had distinct strengths. First, the high prevalence of syphilis and the high risk of congenital syphilis in this population were important to demonstrate the substantial cost benefit of introducing the RPR test. Second, all patients at risk for congenital syphilis were included hence the findings are wholly representative and relatable to other routine programmes in similar settings.

Limitations include that the study was conducted in an urban setting and may hence not be generalizable to other parts of the country. Another limitation is that the study did not look at neonatal outcomes past the initial hospitalization period so late symptoms of congenital syphilis would have been missed. This subject warrants further investigation in future studies. However, since all babies were treated with at least one dose of penicillin, the risk of late onset congenital syphilis is low [22].

The cost and health care benefits of antenatal syphilis screening is well known [24]. Several studies have also described the benefits of using various syphilis testing algorithms during antenatal care in different contexts [25]. There are no studies that have explored the operational costs and benefits for the initial hospital care of newborns of using this type of treatment algorithm.

This study has some important implications. The cost savings are expected to vary as a function of the percentage of delivering women who are both SD Bioline positive and RPR reactive. The higher the percentage of women with past resolved infection the more costs saved with this algorithm. Ideally the risk of congenital syphilis is most greatly reduced by reinforcing antenatal testing and treatment of syphilis; nevertheless implementation of this testing and treatment strategy for infants at risk for syphilis will have several benefits. It could greatly decongest neonatal wards thus freeing up beds for other neonates who may need them and prevent overtreatment and the associated risks for babies born to mothers with syphilis.

## Conclusions

In conclusion, congenital syphilis continues to be a significant morbidity requiring treatment among neonatal populations. In sites where there is high prevalence of syphilis the testing and treatment strategy should appropriately identify and treat those infants at risk for congenital syphilis. This study demonstrates that an algorithm like the one implemented in Castors can help distinguish between infants at high and low risk and treat them accordingly in a low-resource setting. The implementation of an algorithm like this also has significant benefits for the infants in reducing unnecessary risks associated with antibiotic use and hospitalization and for the hospitals in rationalizing expenditure in health care provision.

## Data Availability

The datasets used and/or analysed during the current study are available from the corresponding author on reasonable request.

## Abbreviations

BEmONC: Basic emergency obstetric and newborn care centre
CAR: Central African Republic
CEmONC: Comprehensive Emergency Obstetric and Newborn Care Center
MSF: Medecins Sans Frontieres
MTCT: Mother to child transmission
PCN: Penicillin
RPR: Rapid plasma reagin
